# Utilizing Drug Amorphous Solid Dispersions for the Preparation of Dronedarone per os Formulations

**DOI:** 10.3390/polym15214292

**Published:** 2023-11-01

**Authors:** Afroditi Kapourani, Alexandra-Eleftheria Manioudaki, Konstantinos N. Kontogiannopoulos, Panagiotis Barmpalexis

**Affiliations:** 1 Laboratory of Pharmaceutical Technology, Division of Pharmaceutical Technology, School of Pharmacy, Faculty of Health Sciences, Aristotle University of Thessaloniki, 54124 Thessaloniki, Greece; akapourag@pharm.auth.gr (A.K.); alexeleman@gmail.com (A.-E.M.); kontogik@pharm.auth.gr (K.N.K.); 2 Natural Products Research Centre of Excellence-AUTH (NatPro-AUTH), Center for Interdisciplinary Research and Innovation (CIRI-AUTH), 57001 Thessaloniki, Greece

**Keywords:** Dronedarone, amorphous solid dispersions, Soluplus, sustained supersaturation, molecular dynamics simulations

## Abstract

Dronedarone (DRN), an antiarrhythmic drug, exhibits potent pharmacological effects in the management of cardiac arrhythmias. Despite its therapeutic potential, DRN faces formulation challenges due to its low aqueous solubility. Hence, the present study is dedicated to the examination of amorphous solid dispersions (ASDs) as a strategic approach for enhancing the solubility of DRN. Initially, the glass forming ability (GFA) of API was assessed alongside its thermal degradation profile, and it was revealed that DRN is a stable glass former (GFA III compound) that remains thermally stable up to approximately 200 °C. Subsequently, five commonly used ASD matrix/carriers, i.e., hydroxypropyl methylcellulose (HPMC), povidone (PVP), copovidone (PVP/VA), Soluplus^®^ (SOL), and Eudragit^®^ E PO (EPO), were screened for the formation of a DRN-based ASD using film casting and solvent shift methods, along with miscibility evaluation measurements. SOL proved to be the most promising matrix/carrier among the others, and, hence, was used to prepare DRN ASDs via the melt-quench method. The physicochemical characterization of the prepared systems (via pXRD) revealed the complete amorphization of the API within the matrix/carrier, while the system was physically stable for at least three months after its preparation. In vitro release studies for the ASDs, conducted under non-sink conditions, revealed the sustained supersaturation of the drug for at least 8 h. Finally, the use of attenuated total reflectance (ATR) FTIR spectroscopy showed the formation of a strong molecular interaction between the drug molecules and SOL.

## 1. Introduction

Dronedarone (DRN) is a structural analog of amiodarone with a log octanol/water partition coefficient (LogP) of 6.46 [1,2]. It is prescribed to patients with paroxysmal or persistent atrial fibrillation as an antiarrhythmic drug that works to restore the normal sinus rhythm. DRN is approved as orally administrated tablets for the treatment of atrial fibrillation [3,4]. However, DRN (even in its hydrochloride salt form) is considered to be a poorly water-soluble drug, which has a low solubility in various physiological pH conditions, such as at the gastric (pH 1.2) area (Class II compound according to Biopharmaceutical Classification system) [5,6,7]. Specifically, DRN hydrochloride is practically insoluble in water (0.64 mg/mL) and in buffers such as gastric fluid pH 1.2 and intestinal fluid pH 6.8 (<0.01 mg/mL) [6]. To improve its solubility, the commercially available tablet (i.e., Multaq^®^, Sanofi, France) uses a triblock copolymer of polyethylene−propylene glycol as a solubility enhancer [8]. However, despite this approach, the absolute oral bioavailability of DRN is still limited (approximately 4% under fasting conditions and 15% after food intake). To address this low bioavailability problem, researchers have proposed several alternative formulations including the use of inclusion complexes with cyclodextrins [6,9], micronization [10], formation of pro-liposomes [11], and the preparation of solid dispersions (SDs) [5,12,13].

SDs refer to a category of solid products comprising at least two distinct components, typically a matrix/carrier and a drug [14]. In these SD systems, the drug can be either in crystalline form, which mostly leads to forming API nanocrystals, or in amorphous form, which preferably leads to molecularly dispersed SDs. These latter SD systems comprise the amorphous solid dispersions (ASDs) and display a higher Gibbs free energy, compared with crystalline SDs, thus leading to further enhancement in aqueous solubility and, consequently, to bioavailability [15,16]. Despite this advantage, a significant drawback of ASDs, compared with crystalline SDs, is their physical instability during storage or after solubilization [17,18,19,20,21,22,23,24,25]. Therefore, taking the importance of recrystallization into account, the selection of appropriate matrix/carrier(s) (which are mostly polymers or copolymers) is crucial in order to ensure the physical stability of the system. Incorporating amorphous drugs into the complexed networks of the three-dimensional structures of polymers (or copolymers), with numerous interchain or intrachain links, impedes their molecular mobility. As a result, the chemical potential of the amorphous drug is lowered and simulates that of the crystalline counterpart [26]. In this way, matrix/carriers may prevent the devitrification process of API. In addition, molecular interactions between the API molecules and the matrix/carrier, such as hydrogen bonds (H-bonds), Van der Waals, ionic, and π−π stacking interactions, may also serve as a source of system stabilization [27,28].

Regarding DRN, there have been only a few attempts until now evaluating the use of ASDs for the enhancement of API’s aqueous solubility. Specifically, in the work of Han et al., DRN ASDs were prepared using Soluplus^®^ (SOL; a polyvinyl caprolactam−polyvinyl acetate−polyethylene glycol graft copolymer) as a matrix/carrier via hot-melt extrusion [5]. The results in this study showed that the prepared ASDs exhibited a faster in vitro dissolution profile compared with the marketed, Multaq^®^, drug product. Similarly, Jung et al. prepared DRN-based ASDs using a conventional solvent evaporation method [12]. In this study, two different polymers (namely, hydroxypropyl methyl cellulose (HPMC) and poly(vinyl pyrrolidone) (PVP)) and a copolymer (namely, vinylpyrrolidone-vinyl acetate copolymer (PVP/VA)) were tested, with the results showing enhanced dissolution profiles in all cases, compared with the crystalline API. Despite their significance, it is important to note that in these studies, only preliminary tests on the ASDs were performed, focusing on the successful amorphization of the API (immediately after preparation) and the dissolution performance of the ASDs only under sink conditions. Finally, Kapourani et al. used DRN as a poorly water soluble drug for the evaluation of a new polyester (i.e., poly(propylene succinate)) as a plasticizer in hot-melt extrusion processes [13]. In this study, DRN served as a model drug, and, as a result, the research did not focus on the development and optimization of a DRN-based ASD.

Therefore, despite being limited in number, previous research studies clearly suggest that ASDs could be a promising formulation strategy for enhancing DRN’s aqueous solubility (and consequently oral bioavailability). However, despite the progress made, a considerable amount of work is still needed to fully unlock the potential of new DRN ASD systems. This includes acquiring more profound knowledge of the diverse molecular events occurring during the preparation and storage of DRN ASD’s and attaining a more comprehensive perception on the molecular interplay between the distinct elements within the ASD system. Hence, the goal of the present study was to lay the necessary work to advance the research related to the development of DRN ASDs, focusing, for the first time, on the selection (screening) of suitable matrix/carries, the physical stability during storage, and the presence of molecular interactions between ASDs compounds.

## 2. Materials and Methods

### 2.1. Materials

DRN (as a hydrochloride salt, with purity over 99.5% *w*/*w*), hydroxypropyl methylcellulose (HPMC K4M, AFFINISOL™, Shin-Etzu, Tokyo, Japan), poly(butyl methacrylate-co-(2-dimethylamino) ethyl methacrylate-co-methyl methacrylate (Eudragit^®^ E-PO, EPO, Evonik, Essen, Germany), and hypromellose acetate succinate (HPMCAS, Aquoat^®^ AS-LF, Shin-Etzu, Tokyo, Japan) were kindly given as a gift from Rontis Hellas S.A. (Athens, Greece). SOL, PVP (Kollidon^®^ K12), and PVP/VA (Kollidon^®^ VA64) were obtained from BASF (Ludwigshafen, Germany). All of the other reagents were of analytical or pharmaceutical grade and used as received.

### 2.2. Thermophysical Characterization of DRN

#### 2.2.1. DRN’s Glass Forming Ability (GFA)

A DSC heating−cooling−heating cycle was used to determine and evaluate the correlation between the GFA of DRN [29]. The samples were meticulously weighed (~5 mg) and placed in sealed pans for preparation. These pans were then subjected to a controlled heating and cooling process using a DSC 204 F1 Phoenix heat flux DSC (NETZSCH, Germany). The temperature was raised at a rate of 10 °C/min until it exceeded the API’s melting point. After maintaining this temperature for 3 min to eliminate any prior thermal effects, the samples were gradually cooled at a rate of 10 °C/min until they reached a temperature 10 °C below the glass transition temperature (*T_g_*). Subsequently, they were reheated at a rate of 10 °C/min to a temperature 10 °C above the melting point. A nitrogen flow of 50 mL/min was employed, while the instrument’s temperature and enthalpic calibration was achieved using high purity. All of the tests were carried out in triplicate, and the standard deviations for both temperatures and enthalpies did not exceed 0.8 °C and 2.4 J/g, respectively.

#### 2.2.2. Thermal Stability

To assess DRN’s thermal characteristics, thermogravimetric analysis (TGA) was employed. In this procedure, precise quantities of API (~10 mg) were placed in aluminum pans, which were affixed to the highly sensitive microbalance unit of a Shimadzu TGA-50 thermogravimetric analyzer (Shimadzu Europa GmbH, Duisburg, Germany). The samples were subjected to heating within a nitrogen atmosphere (supplied at a rate of 50 mL/min), with the temperature increasing from 25 °C to 300 °C.

### 2.3. Selection of the ASD Matrix/Carrier

For the selection of the most suitable matrix/carrier, three different methods were employed: (1) based on the miscibility of the components, (2) based on their performance during storage stability (film-casting), and (3) based on their performance during solubilization (solvent-shift).

#### 2.3.1. Miscibility Evaluation

The miscibility of DRN and the matrix/carriers was calculated on a theoretical level using the Hansen solubility parameters, computed based on the Hoftyzer−Van Krevelen group contribution technique:

(1) $${\;\;\;\delta }_{t}=\sqrt{\delta_{d}^{2}+\delta_{p}^{2}+\delta_{h}^{2}}$$

where *δ_d_*, *δ_p_*, and *δ_h_* are the partial solubility parameters for intermolecular dispersion, as well as polar and hydrogen bonding forces, respectively.

In addition to the theoretical calculations, the miscibility of the binary API-matrix/carrier system in the melt was also evaluated experimentally using hot-stage polarized light microscopy (HSM). In particular, binary physical mixtures (PMs) of DRN and matrix/carriers (at a ratio of 30/70 *w*/*w*) were heated to 160 °C until the complete melting of both components, on a Linkam THMS600 heating stage (Linkam Scientific Instruments Ltd., Surrey, UK) attached to an Olympus BX41 polarized light polarized on a microscope (PLM, Olympus, Tokyo, Japan).

#### 2.3.2. Film Casting Film Method

The identification of the most promising matrix/carrier, based on the performance of ASD during storage, was accomplished using the film-casting method. Initially, 0.05 g of both DRN and the selected matrix/carriers were dissolved together in appropriate solvent quantities (ranging from 3 to 5 mL) such as ethanol, acetone, methanol, and DMSO. A 50/50 *w*/*w* of API to carrier was deliberately chosen to induce the drug’s recrystallization process. Subsequently, the prepared API/polymer solutions were dispensed onto standard microscopy slides and subjected to vacuum oven drying at 45 °C (11 psi) overnight, forming a thin solventless film. Pure DRN was also used for comparative analysis. After solvent evaporation, ASD formation was confirmed using polarized light microscopy (PLM) with a PRIOR microscope (Prior Scientific Instruments Ltd., Cambridge, UK). These samples, placed in desiccators, were maintained at 30 °C and 60% relative humidity (RH) and the microscopic slides were examined (with PLM) at several timepoints. Specifically, photographs of the samples were taken every five days.

#### 2.3.3. Solvent-Shift Method

The selection of the most suitable matrix/carrier was also contingent on ASD’s performance during the solubilization process. To assess this, each matrix/carrier’s ability to prevent the precipitation of DRN in a hydrochloric acid buffer (pH 1.2) was examined using the solvent-shift method, based on a modified method adopted from a previously published study [30,31]. The dissolution tester maintained conditions at 37 ± 0.5 °C with a stirring speed of 100 rpm using a paddle. Initially, about 5 mL of a 10% *w*/*v* DRN solution in methanol was gradually introduced into a dissolution medium comprising 500 mL of the chosen buffer (with or without the polymer). In cases where the buffer contained a polymer, the polymer concentration was 10 mg/mL. At specified intervals (5, 10, 15, 30, 45, 60, and 90 min), approximately 3 mL aliquots were withdrawn and subsequently centrifuged at 17,586× *g* for 3 min. The resulting supernatant containing DRN was then filtered through a 0.20 μm hydrophilic membrane (Sartorius, Germany, Model Minisart RC 25) and diluted with methanol prior to analysis using a UV/VIS spectrometer (Shimadzu, Kyoto, Japan) at a wavelength of 293 nm. The quantitative assessment of the test samples was conducted using a methanolic calibration curve with good linearity (R^2^ = 0.999) in the DRN concentration range of 5–50 μg/mL.

### 2.4. Preparation of Amorphous and ASD Samples

ASDs were prepared via the melting quench-cooling approach. In short, DRN or DRN−carrier mixtures of approximately 1 g in total (10/90, 20/80, and 30/70 *w*/*w* of API to matrix/carrier) were heated on aluminum pans at 180 °C on a heating plate for 10 min, until a melt dispersion was observed. The samples were then quenched at −30 °C in a freezer. The ASDs were gently ground and then sieved through 150−180 μm before further use.

### 2.5. Physicochemical and Thermophysical Characterization of ASDs

#### 2.5.1. Differential Scanning Calorimetry (DSC)

Similar to the DSC procedure followed for the evaluation of GFA and GS, ASD samples of DRN and the most promising matrix/carrier with 10, 30, 50, 70, and 90% *w*/*w* of drug were prepared and subsequently heated (20 °C/min) up to 200 °C, cooled at a rate of 20 °C/min to −10 °C, and reheated (20 °C/min) up to 200 °C. The selection of the temperature range used was based on the melting point and the glass transition temperature (*T_g_*) of DRN, which are approximately 141 °C and ~51 °C, respectively [13]. The graph of peak areas (or shifts) with temperature was constructed and used to compare *T_g_* and *T_melt_*.

#### 2.5.2. Powder X-ray Diffractometry (pXRD)

pXRD is used to confirm the presence of amorphously dispersed API in the prepared samples [32]. Specifically, pXRD diffractograms of DRN, SOL, and the ASDs after preparation and after three months of storage at 25 °C and 60%RH, were collected using a D2 PHASER XRD diffractometer (Bruker AXS, Karlsruhe, Germany) using Cu radiation at 30 kV and 100 mA. All of the samples (quantity of each sample equal to 0.5 g) were scanned from 5 to 40°, 2θ.

#### 2.5.3. Molecular Interactions

Experimentally: Molecular interactions were assessed using attenuated total reflectance Fourier transform infrared (ATR-FTIR) spectroscopy. ATR-FTIR spectra spanning from 700 to 4000 cm^−1^ were collected for DRN, the most promising carrier, and their ASDs. A Shimadzu IR-Prestige-21 FTIR spectrometer coupled with a horizontal Golden Gate MK-II single-reflection ATR system from Specac (Kent, UK), equipped with a ZnSe lens, was used. The analysis involved 64 scans over the specified wavenumber range at a resolution of 4 cm^−1^, with each sample subjected to appropriate background subtraction [33].

Theoretically: Theoretical insights into the formation of molecular interactions were collected through molecular dynamics (MD) simulations. The initial molecular structures of DRN and the matrix/carrier were generated using XenoView (available online at http://www.vemmer.org/xenoview/xenoview.html (accessed on 30 June 2023). Energy minimization of the initial structures was executed via the pcff_d force field, employing the steepest descent algorithm. Here, 1.00 × 10^−4^ kcal/mol was set as a tolerance limit, while 10 Å was set as the maximum displacement per iteration. Amorphous assemblies were constructed utilizing XenoView’s amorphous builder, while a multistep equilibration protocol was employed to ensure the formation of homogeneously mixed simulation boxes [34]. A 10.0 ns simulation was conducted using the NPT ensemble at 1 atm and 25 °C (cutoff radius of 10 Å, spline distance of 1 Å, Berendsen thermostat and barostat, and a time step of 1 fs). Van Gunsteren and Mark criteria were employed for the validation of the model [35]. The trajectory of the last 5 ns from the MD simulation run was assessed for the calculation of the radial distribution function, *g*(*r*), based on the following:

(2) $$\;\;g(r)=\frac{\langle V\Sigma_{i\neq j}\delta \left(r-\left|r_{Ai}-r_{Bi}\right|\right)\rangle }{\left({Ν}_{A}N_{B}-N_{AB}\right)4\pi r^{2}dr}$$

where *A* and *B* are specific atoms; *V* is the system volume; *N_A_* and *N_B_* are particle number of atoms *A* and *B*, respectively; *N_AB_* are the number of particles belonging to atom *A* and atom *B* simultaneously; and *r_Ai_* and *r_Bj_* denote the positions of particle *i* of atom *A* and particle *j* of atom *B*, respectively.

### 2.6. Saturation Solubility and Dissolution Studies

#### 2.6.1. DRN’s Crystalline Saturation Solubility Determination

The saturation solubility of crystalline raw DRN was determined in a pH 1.2 hydrochloric acid buffer. Briefly, an excess amount of DRN was inserted into 50 mL of the buffer at 37 °C and stirred for 48 h. The sample was filtrated (using a 0.20 μm Minisart RC 25 syringe filter) and the concentration of DRN was measured via a UV−VIS spectrometer (Pharma Spec UV-1700, Shimadzu Europa GmbH, Duisburg, Germany) at 293 nm. The equilibrium solubility, *Cs*, of DRN at pH 1.2 was found to be 0.01 mg/mL.

#### 2.6.2. Non-Sink Condition Dissolution Studies

Dissolution experiments of the prepared ASDs, along with the neat amorphous DRN, were conducted using an apparatus II (paddles) dissolution tester (PT-DT7 Pharma Test AG, Hainburg, Germany) with 100 mL of Simulated Gastric Fluid (pH 1.2) at a constant temperature of 37 °C and a paddle rotation rate of 100 rpm. All of the samples were tested as particles, while the size of ASDs and the pure DRN used in the dissolution study was controlled in the range of 150–180 μm using a suitable set of stainless-steel sieves. Non-sink conditions were deliberately maintained to induce the typical supersaturation seen in the gastrointestinal (GI) tract, leading to nucleation and crystallization events. The deviation from sink conditions was quantified using a dimensionless sink index (SI) of 0.1. SI is defined as the ratio of the product of Cs·V to dose, where Cs is the saturation solubility of the crystalline drug, V is the volume of dissolution medium, and dose is the total amount of the drug in the sample. Taking into account the saturation solubility of DRN and the use of a 100 mL dissolution medium, it is evident that the drug dosage applied was 10 mg. At predetermined intervals (i.e., 5, 10, 15, 30, 45, 60, 90, 120, 180, 240, 300, 360, 420, and 480 min), 3.0 mL aliquots were withdrawn and replaced with fresh dissolution medium. These aliquots were immediately filtered through a 0.1 μm Whatman filter and diluted with MeOH to prevent drug crystallization after sampling. Drug concentrations were determined spectrophotometrically using a UV−VIS spectrometer (Pharma Spec UV-1700, Shimadzu Europa GmbH, Duisburg, Germany) at 293 nm. All of the experiments were conducted in triplicate.

### 2.7. Stability Studies

Amorphous DRN and ASDs (~2 g) with varying ratios of API to carrier (10/90, 20/80, and 30/70 *w*/*w*) were placed in open vials within desiccators at elevated humidity levels (60% RH) at room temperature. After three months, all samples were subjected to an analysis to assess potential alterations in the API’s physical state using pXRD.

## 3. Results and Discussion

### 3.1. Thermophysical Properties of DRN

Before progressing with the development of ASDs, it is essential to assess the inherent characteristics of API, particularly those concerning its physical (especially amorphous phase) and thermal stability.

#### 3.1.1. GFA Classification of DRN

**Figure 1:**
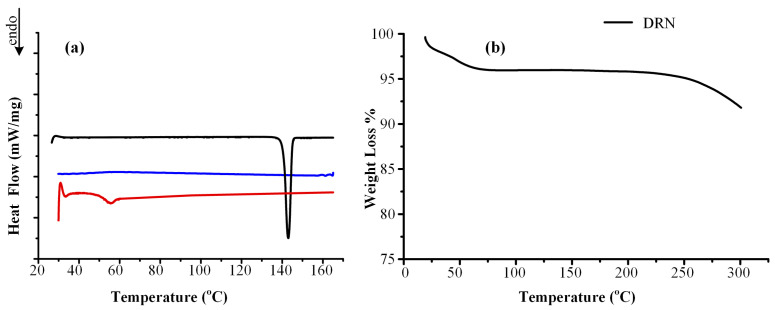
(**a**) DSC thermograms of DRN during the heating (1st run, black)–cooling (blue)–heating (2nd run, red) cycle for the determination of GFA. (**b**) TGA-based weight loss of DRN (thermal degradation).

GFA serves as a measure of the ease with which the vitrification process occurs for a drug substance. In the present study, the GFA of the API was evaluated via solidification from its melt during a DSC heating–cooling–heating cycle according to Baird et al. [29]. More specifically, according to this particular method, compounds can be categorized into three distinct classes (Class (I), Class (II), Class (III)) based on whether crystallization is observed or not during a heating–cooling–heating cycle, as determined by DSC. Figure 1a provides a summary of the DSC thermograms obtained to assess the glass-forming ability (GFA) of DRN.

The initial heating phase revealed only a melting endotherm for the API, characterized by a melting temperature (*T_melt_*) at 144.0 °C and an enthalpy of fusion (Δ*H_f_*) of 95.06 J/g. Upon cooling, no thermal events were observed, while the subsequent reheating revealed only a glass transition temperature (*T_g_*) at 55.5 °C. Consequently, the results indicate that DRN falls into the category of a stable glass former, belonging to the class III of glass-forming ability (GFA) compounds according to Baird et al. [29].

#### 3.1.2. Thermal Stability of DRN

In the subsequent phase, it is significant to examine the stability of the neat DRN under thermal treatment. This evaluation is critical in order to assess the suitability of manufacturing processes that apply heating for the preparation of ASDs. The TGA thermogram, illustrated in Figure 1b, indicates that DRN remained stable up to ~225 °C. This finding is in agreement with the results presented in previously published studies [6,13]. A small percentage of water moisture was also detected, revealed by the mass loss of ~3% *w*/*w* in the TGA thermogram. Further elevation of temperature (beyond 225 °C) showed the pronounced reduction in DRN weight, signifying the fast degradation of the drug. Consequently, it is reasonable to assume that API maintains thermal stability up to around 220 °C. Therefore, manufacturing processes utilizing heating temperatures within the range of DRN’s melting point (i.e., 140–180 °C) can be safely employed for manufacturing ASDs.

### 3.2. Selection of the ASD Matrix/Carrier

Following the assessment of DRN’s thermal and GFA attributes, a variety of widely used polymers and copolymers (namely, HPMC, PVP, coPVP, SOL, and EPO) were examined to identify the best ASD matrix/carrier(s). In order to achieve this, three distinct methods were employed: (1) miscibility evaluation method; (2) film-casting method, which assesses the ASD system’s performance regarding recrystallization during storage; and (3) solvent-shift method, which examines the system’s performance during the solubilization process.

#### 3.2.1. Miscibility Evaluation Results

A pivotal factor determining the formation of an ASD is the miscibility of the drug with the matrix/carrier. If there is immiscibility among these components, phase separation becomes thermodynamically favorable. Therefore, in this study, the miscibility of DRN with the examined matrix/carriers was assessed using two approaches: one theoretical, employing Hansen solubility parameters, and another experimental, utilizing HSM.

Hansen solubility parameters (HSPs): Table 1 displays the HSPs for DRN, with the calculated total solubility parameter (*δ_t_*) for API determined as 18.5 MPa^1/2^. Similarly, employing the same group contribution method, the *δ_t_* values for all of the tested matrix/carriers were as follows: 19.9, 22.4, 21.5, 21.8, and 20.6 MPa^1/2^ for HPMC, PVP, PVP/VA, SOL, and EPO, respectively [31,36,37].

In general, the difference in *δ_t_* values between two components, denoted as Δ*δ_t_* for the drug and matrix/carrier, serves as an indicator of their miscibility. API-matrix/carrier combinations are considered to be immiscible when the absolute Δ*δ_t_* values exceed 10 MPa^1/2^, miscible when Δ*δ_t_* is below 7 MPa^1/2^, and are likely to yield a glassy solid solution when Δ*δ_t_* falls below 2 MPa^1/2^ [38]. Hence, it seems that DRN is miscible with all of the tested matrix/carriers as the Δ*δ_t_* of 1.4, 3.9, 3.0, 3.3, and 2.1, were calculated for the mixtures of the API with HPMC, PVP, PVP/VA, SOL, and EPO, respectively. Interestingly, although DRN is miscible with all of the tested matrix/carriers, it seems that, at least based on the HSPs, API may form glassy solid solutions only with HPMC.

**Figure 2:**
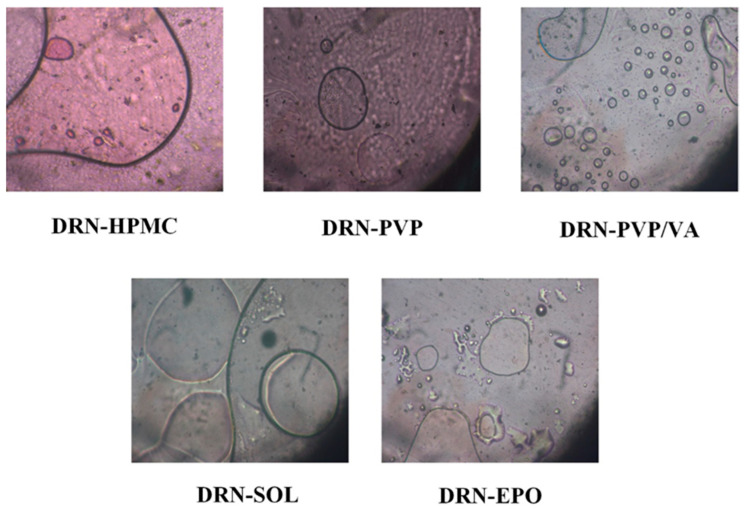
PLM photograph depicting the melt miscibility of DRN with the tested matrix/carrier at 160 °C.

HSM: The miscibility of the two binary ASD components (at the melt state) was also evaluated experimentally via HSM. According to the obtained results, illustrated in Figure 2, DRN and all of the tested matrix/carriers exhibited full miscibility. No discernible melting or separation zones were observed between the binary API/(co)polymer components. Consequently, these outcomes validate the earlier theoretically derived findings (utilizing HSPs) and confirm the reliability of both approaches in predicting the miscibility of the two components.

#### 3.2.2. Film-Casting Method

**Figure 3:**
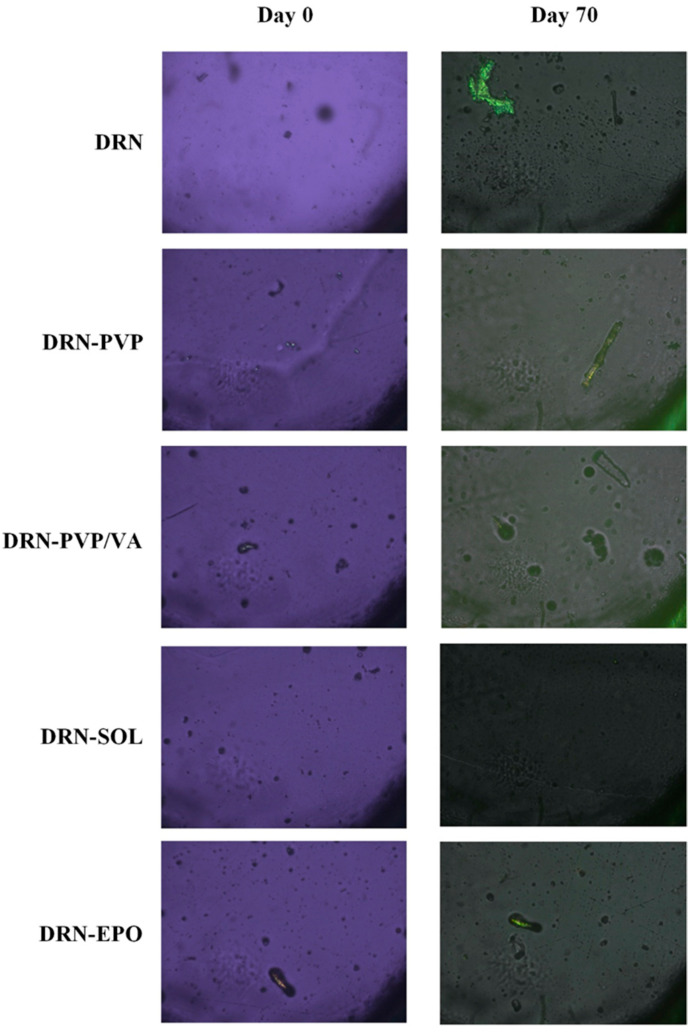
Selection of DRN’s most suitable matrix/carrier based on the film-casting method.

In this method, the screening of the matrix/carriers involves visually inspecting ASD films created from binary mixtures of DRN and each matrix/carrier. This assessment occurs immediately after preparation and subsequent storage under 30 °C and 60% RH, respectively, for several days. The matrix/carrier that demonstrates the most pronounced inhibition of API’s crystallization was chosen as the most suitable matrix/carrier for the formulation of ASDs according to this method. Figure 3 illustrates the PLM photographs from the neat DRN and the DRN-matrix/carrier films.

Regarding the neat DRN, the results revealed that following the preparation of the glassy film, the amorphous API remained stable until ~day 70, after which a slight recrystallization was observed. These findings confirm that the drug is indeed a GFA III compound, forming a relatively stable glass without the need for a crystallization inhibitor. However, as evident from the images in the same figure, API recrystallization began after approximately two months of storage. Therefore, in order to ensure that a DRN-based amorphous formulation remains stable throughout the intended shelf-life of the product, it is crucial to include a suitable matrix/carrier that will serve as a recrystallization inhibitor. Results concerning the DRN-matrix/carrier mixtures indicate that in the case of PVP/VA and EPO, there is a slight API recrystallization even at day zero, which is immediately after the preparation of the ASD films. This suggests that the presence of these two matrix/carriers not only fails to stabilize the amorphous API, but, on the contrary, accelerates recrystallization compared with the neat API alone. In the case of PVP and SOL, no API crystals were observed at zero time. However, after 70 days of storage, only in the case of SOL did the API remain completely amorphous, as small API crystals were observed for DRN-PVP binary films. This suggests that SOL is the only matrix/carrier, among those tested in the present study, that effectively inhibited the recrystallization process of API. Therefore, at least based on the film-casting method, SOL was identified as the most suitable matrix/carrier for the formation of a DRN ASD system.

#### 3.2.3. Solvent-Shift Method

**Figure 4:**
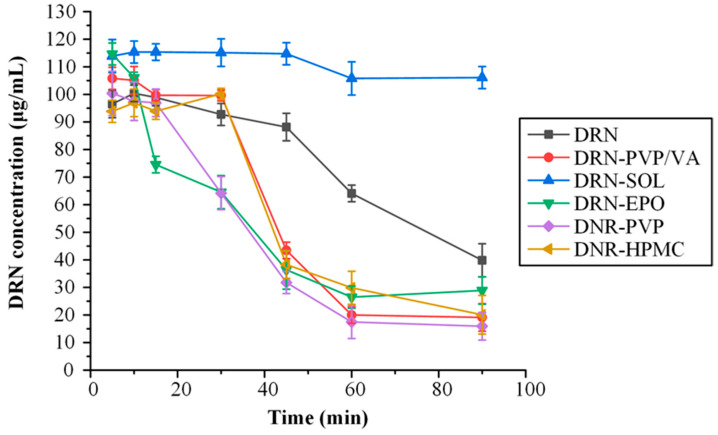
Selection of DRN’s most suitable matrix/carrier based on the solvent shift method.

This method assessed the capacity of a matrix/carrier to prevent the precipitation of the API during solubilization. Figure 4 shows the changes of DRN concentration vs. time in the presence or absence of the tested matrix/carriers.

The findings indicate that SOL surpassed all other matrix/carriers, demonstrating a superior ability to sustain higher concentrations of DRN in the dissolution medium. Interestingly, it appears that all the other examined matrix/carriers, except for SOL, led to lower levels of DRN concentration in the dissolution medium, compared with the neat API. This suggests that the presence of these (co)polymers induces the precipitation of the API from the solute state, resulting in an inferior performance even when compared with the neat API alone.

Therefore, it seems that SOL can be considered as the most suitable matrix/carrier for the creation of DRN-based ASDs.

### 3.3. Preparation and Evaluation of ASDs

Following the identification of SOL as the most suitable copolymer for inhibiting the re-crystallization of DRN during storage and solubilization and confirming their miscibility, DRN-SOL ASDs were produced at several drug-to-copolymer ratios (i.e., 10/90, 20/80, and 30/70 *w*/*w* of API to matrix/carrier). Glassy and brittle solids were acquired in all cases. The obtained ASDs underwent thorough analysis to assess their physical state, in vitro supersaturation, and the development of molecular interactions.

#### 3.3.1. Evaluation of DRN’s Physical State

**Figure 5:**
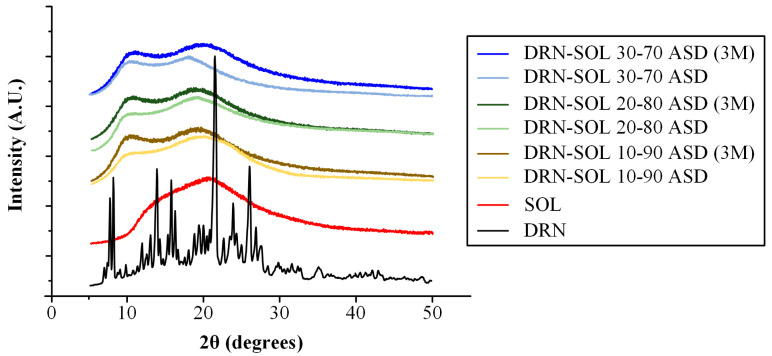
pXRD diffractograms for the raw materials (LUT and PVP) and the LUT-PVP (30/70 *w*/*w*) ASDs immediately after preparation and after storage for three months (3M).

Figure 5 illustrates the pXRD diffractograms of the neat DRN and the ASDs prepared at the several weight ratios.

The diffractogram of DRN shows several characteristic peaks at 2θ of 7, 8, 13, 15, 16, 21, 23, and 26°. The obtained diffractogram corresponds to DRN form I crystals [5,39]. Regarding the ASDs, all characteristic peaks of DRN were absent, signifying the amorphous dispersion of the drug within the SOL matrix/carrier. Furthermore, changes in the physical state of the ASDs were assessed after storage for three months. The diffractograms presented in the same figure reveal that, in all cases (across all drug-to-copolymer ratios), no recrystallization of the API occurred during storage, as only an amorphous halo is seen for all ASDs. Therefore, it can be concluded that DRN remains stable in all of the formed ASDs, at least for the three months, tested in the present study.

#### 3.3.2. In Vitro Supersaturation Studies

In the next step of the present study, the in vitro supersaturation of the DRN-SOL ASD formulations was investigated. Typically, in vivo drug solubilization results in the common occurrence of supersaturation buildup, which often leads to recrystallization and suboptimal bioavailability. Modern approaches to amorphous drug formulation recognize that, in ASDs, it is not only important for the drug to maintain its amorphous stability during storage, but it is equally crucial to preserve its supersaturation upon solubilization.

Creating and sustaining a metastable supersaturated dissolved state is a beneficial approach for enhancing the gastrointestinal absorption of poorly water-soluble drugs, considering the kinetic nature of dissolution and the thermodynamic property of solubility. This strategy involves two crucial steps known as the “spring and parachute” effects. In the initial step (spring), a thermodynamically unstable, supersaturated solution of the drug is generated. This can arise from a higher energy form of the compound, like the amorphous form with its increased Gibbs free energy, or from the inclusion of a solubility enhancer. During the second step (parachute), the drug dissolves at a high and metastable supersaturated level, and preserves this state for an extended duration. This is primarily accomplished using specific additives or inherent properties of the matrix/carriers themselves. In both instances, these elements act as precipitation inhibitors, effectively slowing down the nucleation rate and recrystallization process of the API following its solubilization in the dissolution medium.

**Figure 6:**
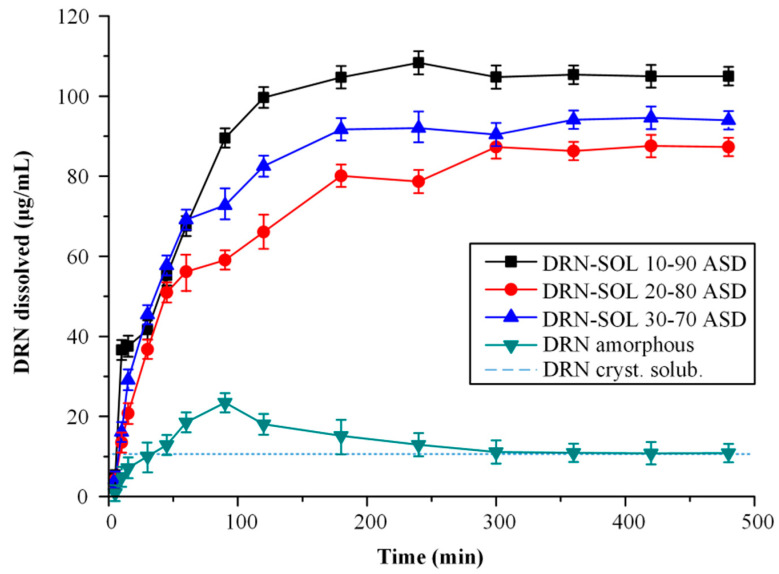
In vitro supersaturation profiles of amorphous DRD and DRN-SOL ASDs at various weight ratios (the dashed line depicts the saturation solubility of DRN in the dissolution medium).

Figure 6 illustrates the in vitro dissolution profiles of the DRN-SOL ASDs compared with the neat amorphous API, along with the saturation solubility line of the crystalline (form I) DRN in the selected dissolution medium (cyan dashed line). As mentioned in Section 2.6.1, the equilibrium solubility of DRN at pH 1.2 was determined to be 0.01 mg/mL.

Regarding the amorphous DRN, the findings reveal an initial rise in release followed by a swift decline, indicating that the supersaturation of the API resulted in its recrystallization. This behavior is typical for amorphous drugs, displaying a spring-like supersaturation profile in the absence of a precipitation inhibitor. In this scenario, the initial high degree of supersaturation triggers drug nucleation, which subsequently leads to API recrystallization. In this particular case, the recrystallization process led in the formation of DRN crystals, likely of form I, given that the concentration level at which the amorphous API reached an equilibrium state matched the saturation solubility of the form I crystals.

On the contrary, the results for all of the prepared ASDs indicated the sustained supersaturation of the API within the dissolution medium. In particular, the utilization of SOL yielded a significantly heightened “spring” effect compared with the pure amorphous API, indicating that the chosen matrix/carrier had the capacity to further enhance the solubility of API. This enhancement can be attributed to SOL’s role to act as a solubility enhancer [40,41]. As previously mentioned, SOL is a polyvinyl caprolactam−polyvinyl acetate−polyethylene glycol graft copolymer, employed as an amphiphilic polymeric solubilizer [42,43]. With both hydrophilic and hydrophobic chains present in its structure, SOL can form micelles even at very low concentrations in aqueous solutions [41], consequently improving the solubility of poorly water-soluble drugs like DRN. In addition, as seen from the obtained dissolution profiles, SOL led to a notable delay in the drug’s supersaturation, leading to sustained supersaturation. This can be ascribed to the existence of hydrophobic chains in SOL’s copolymer structure, which effectively function as precipitation inhibitors, hindering the recrystallization process of API after its solubilization in the dissolution medium [44].

Therefore, it seems that the utilization of SOL resulted in a significantly improved dissolution/supersaturation profile for DRN. It is noteworthy that the overall enhancement in supersaturation correlated with the concentration of the selected matrix/carrier, with higher weight ratios of SOL, leading to greater supersaturation. This observation further supports the assumption that SOL functions as both a solubility enhancer and a precipitation inhibitor, and its impact on both processes is intensified with an increase in its concentration.

#### 3.3.3. Molecular Interactions

Molecular interactions could be partially accountable for the performance, encompassing physical stability and sustained supersaturation, of the formulated ASDs. Therefore, to assess the existence of these interactions and their impact on the properties of the DRN-SOL ASDs, ATR-FTIR spectroscopy was utilized complemented by MD simulations.

##### ATR-FTIR

**Figure 7:**
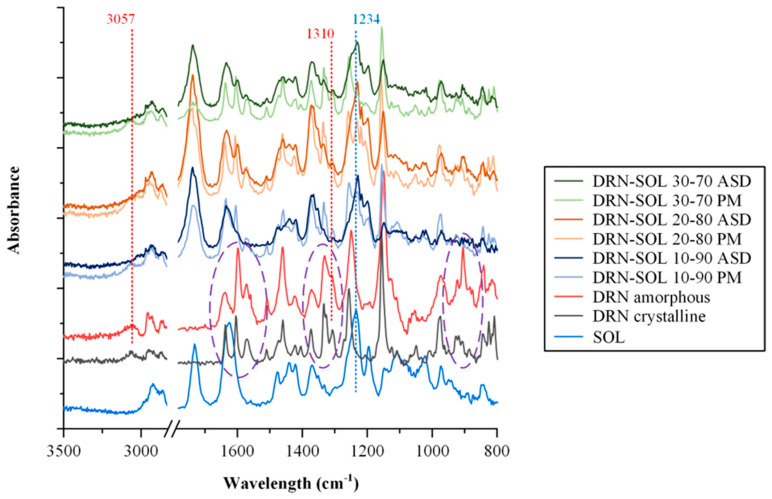
ATR−FTIR spectra of the raw materials (crystalline DRN and SOL), the neat amorphous DRN, and the DRN-SOL ASDs at various weight ratios.

Figure 7 depicts the obtained spectra for the neat DRN (both the crystalline and amorphous API), the neat matrix/carrier (SOL), and the DRN-SOL physical mixtures (PMs, prepared via a mortar and pestle) and ASDs.

Regarding the neat crystalline DRN, the recorded ATR-FTIR spectrum showed all characteristic FTIR peaks [6] located at 3057 cm^−1^ due to sulfonamide NH stretching vibrations, 2958 cm^−1^ due to the aromatic C-H stretching vibrations, 1637 cm^−1^ due to C=O stretching vibrations, and 1310 and 1151 cm^−1^ due to the SO_2_ stretching. As for the differences between the crystalline and the amorphous API, the latter showed several changes in the obtained ART-FTIR spectra, with shifts and new peaks arising mainly in the regions of 1700 to 1500 cm^−1^, 1350 to 1110 cm^−1^, and 900 to 600 cm^−1^ corresponding to the C=O, the −SO_2_, and the aromatic ring stretching regions, respectively (all depicted with purple dashed circles in Figure 7). Concerning the pure matrix/carrier, the observed ATR-FTIR spectrum of SOL displayed various distinctive vibrational peaks, with the most prominent ones occurring at 2913 cm^−1^ (associated with the stretching of −C-H bond), 1732 cm^−1^ (related to the ester −C=O stretching), 1625 cm^−1^ (corresponding to −C(O)N stretching), and 1234 cm^−1^ (associated to the C-O-C ether groups stretching vibrations).

Examining the acquired ATR-FTIR spectra of DRN-SOL, distinct variations between the PMs and their corresponding ASDs became apparent. Specifically, for all PMs, the gathered spectra represented the combination of crystalline DRN and SOL. These observations suggest that no molecular interactions between the two components took place during the physical blending of the components. This outcome aligns with expectations, as the physical mixing process typically does not induce alterations in the physical state or foster any molecular interactions between the two components. On the contrary, the ATR-FTIR spectra of the corresponding ASDs exhibited notable differences in comparison with their PM counterparts. Specifically, no peaks corresponding to the crystalline drug were recorded, as the spectra for all ASDs represented the combination of the respective matrix/carrier and the amorphous API. Hence, it seems that in all of the tested drug to matrix/carrier concentrations, no traces of crystalline API remained after the preparation of the ASDs, a finding that agrees with the previously presented pXRD results. Interestingly, upon closer examination of the obtained spectra, it is evident that in the case of ASDs, the peak at 3057 cm^−1^, corresponding to the sulfonamide NH stretching vibrations of the API, observed in both the neat crystalline and neat amorphous API, is absent. This absence indicates that the NH of the drug is probably involved in the formation of molecular interactions with the matrix/carrier in the prepared ASDs. In addition, the peak corresponding to the C-O-C vibrations of SOL, located at 1234 cm^−1^, is shifted to lower wavelengths in the case of ASDs, indicating that molecular interactions, likely in the form of hydrogen bonds (H-bonds), are possibly forming between the NH groups of the API and the ether oxygens of the matrix/carrier. Also, a similar red shift (i.e., a shift towards longer, or else lower, wavenumbers) is observed for the peaks corresponding to the −SO_2_− stretching vibrations of the API (located initially at 1310 cm^−1^), indicating the formation of other molecular interactions as well.

Hence, in order to explore further into the nature and characteristics of these molecular interactions, MD simulations were conducted.

##### MD Simulations

**Figure 8:**
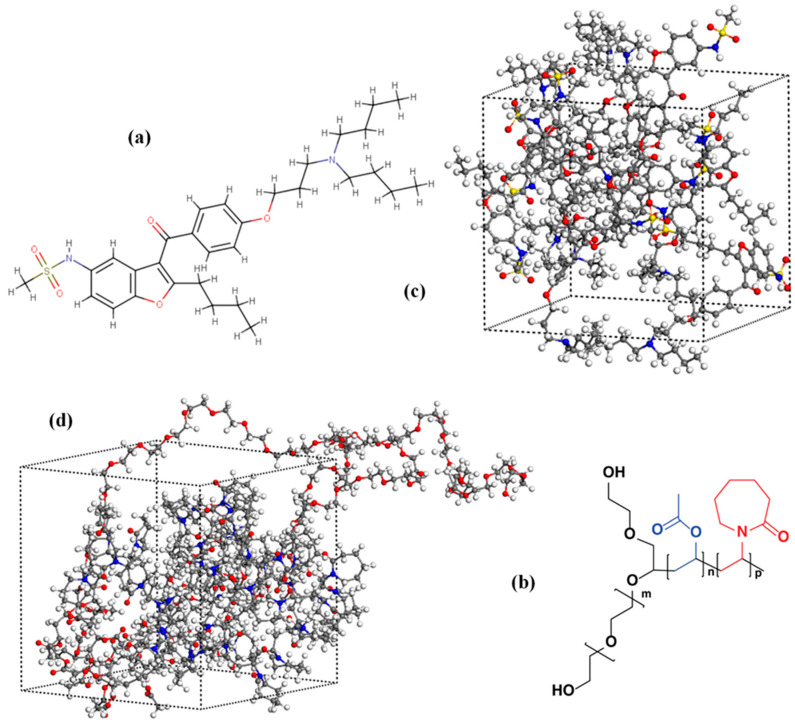
(**a**) Chemical structure of DRN; (**b**) chemical structure of SOL; (**c**) amorphous MD-simulation boxes containing 10 DRN molecules; (**d**) and a SOL chain (hydrogen atoms are depicted with white, carbon with grey, oxygen with red, nitrogen with blue, and sulfur with yellow).

**Figure 9:**
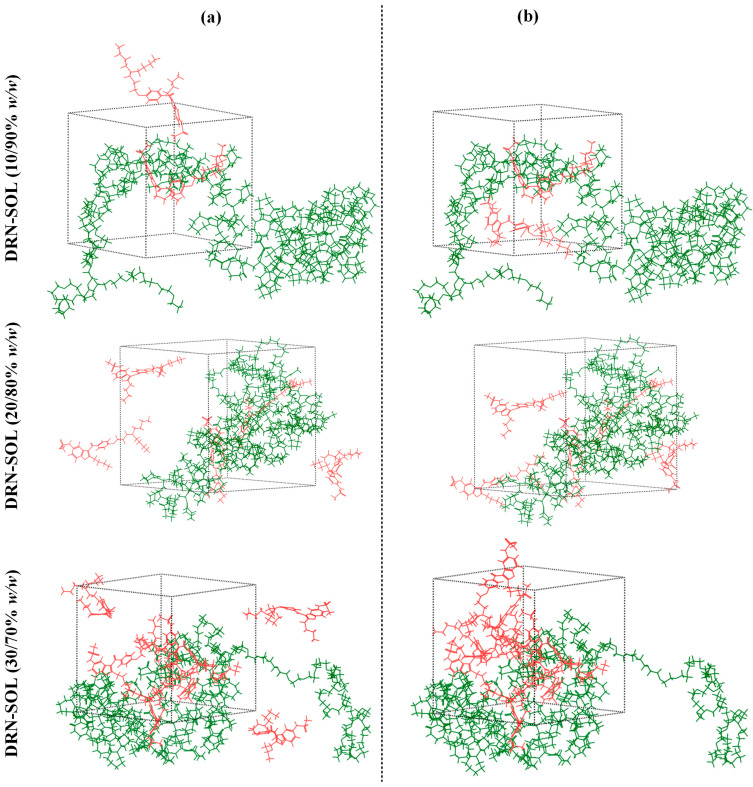
(**a**) MD simulation boxes before (**b**) and after the equilibration protocol (SOL is depicted with green; DRN is depicted with red).

Figure 8 displays the chemical structures of DRN and SOL, as well as their amorphous cell configurations. Figure 9 shows the amorphous assemblies of the DRN-SOL solid dispersions at various drug-to-matrix/carrier concentration ratios before and after the implementation of the equilibration protocol. The procedure followed led to the formation of amorphous structures, wherein the API was more uniformly dispersed in the matrix/carrier, compared with those that did not undergo these equilibration cycles.

Taking a closer look at the structure of DRN, it can be concluded that the API comprises of one H-bond donor (that is the hydrogen in the sulfonamide NH) and several H-bond acceptors (namely, the oxygens in the carbonyl, ether, and sulfur dioxide groups). Similarly, as shown in Figure 8, SOL incorporates numerous H-bond sites (both acceptors and donors). Consequently, it is anticipated that the two compounds will engage in the formation of several H-bonds during the creation of ASDs. Hence, in this study, the extended MD-simulation trajectories of the diverse amorphous assemblies were scrutinized regarding the development of intra- and inter-molecular H-bonds.

**Figure 10:**
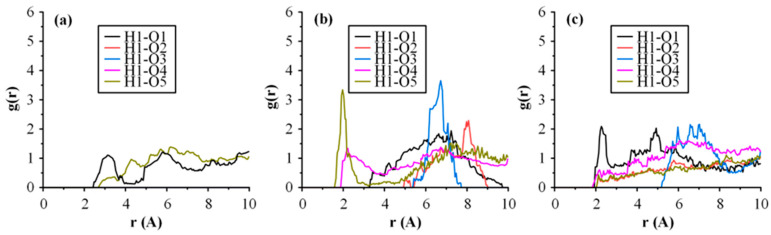
(**a**) Radial distribution functions, *g*(*r*), of intermolecular interactions for the DRN-SOL ASDs at 10/90; (**b**) 20/80; and (**c**) 30/70 *w*/*w* DRN to SOL ratios.

In the case of neat raw materials, MD simulations revealed that H-bonds were formed between the various API or matrix/carrier H-bond acceptors and donors. Additionally, in the case of the ASDs, intermolecular H-bonds were detected between the -NH hydrogen of the API (H1) and the −SO_2_ (O1), −C=O (O2), and −COC− (O2) oxygens of other DRN molecules, or the H-bond acceptors (that is the ether and carbonyl oxygens (O4 and O5) of the polyethylene glycol and the polyvinyl caprolactam and polyvinyl acetate monomers, respectively) of SOL. Hence, to comprehend the nature and strength of all these interactions, the radial distribution function, *g*(*r*), was calculated (Figure 10).

Typically, donor−acceptor distances below 2.5 Å signify the formation of strong interactions between two atoms, while distances falling between 2.5–3.2 Å and above 3.2 Å indicate the formation of moderate and weak (primarily electrostatic) interactions, respectively. In the case of DRN-SOL 10/90 *w*/*w*, the results show the presence of a *g*(*r*) peak at 3.06 Å, corresponding to the formation of moderate intermolecular interactions between the API molecules (that is between the -NH hydrogen, H1, and the sulfur dioxide oxygen, O1, of the API). As the weight ratio of the drug increases, a new strong *g*(*r*) peak is recorded at 1.95 Å between the H1 proton of the API and the −C=O oxygens of SOL (see Figure 10b corresponding to 20/80% *w*/*w* DRN to SOL ratio). This peak indicates the formation of strong intermolecular interactions, probably in the form of H-bonds, between the API and the matrix/carrier molecules. Interestingly, this interaction between the API and SOL seems to disappear with the further increase in API content (that is at 30/70 *w*/*w* in Figure 10c). In this case, a strong intermolecular bond between the API molecules is formed (evident by the presence of a strong H1-O1 *g*(*r*) peak), indicating that with the increasing drug content, DRN seems to form new homonuclear interactions with itself. This may eventually result in the development of a separate drug amorphous region, which could eventually trigger DRN’s recrystallization during stability testing. However, according to the stability data obtained, this presumed drug-rich amorphous region has not caused any API recrystallization for at least three months. Finally, it is important to note that no H-bonds were observed between DND’s H-bond acceptors and SOL’s H-bond donors. This may be attributed to the fact that SOL only possesses two H-donor sites, which are the terminal OH groups of the copolymer, and, hence, due to the limited availability of these H-donor sites within the SOL chains, it is improbable that they significantly contribute to the formation of H-bond interactions in the DRN-SOL ASDs.

## 4. Conclusions

The development of new orally administered antiarrhythmic drug formulations is essential to address the limitations associated with current options, aiming for improved efficacy, safety, and patient compliance in the management of cardiac arrhythmias. However, the formulation of poorly water-soluble antiarrhythmic drugs, such DRN, possesses significant challenges related to the limited drug absorption, due to poor aqueous solubility and permeability. To address these limitations, formulation scientists focus on improving the oral bioavailability of such API by enhancing their solubility and dissolution rates through various techniques, such as ASDs. In this context, the present study was able to advance the research on the formation of a suitable DRN-based ASD and to provide insights into its unique properties by unraveling the mechanisms that contribute to its performance. Among the various matrix/carriers that were evaluated, SOL proved to be the most suitable for the preparation of DRN-based ASDs, as it was miscible with the API (both in the melt and at storage temperature) and was able to effectively inhibit its re-crystallization for several months. The in vitro release evaluation for the prepared DRN-SOL ASDs showed an improvement in API’s kinetic solubility and the sustained supersaturation of the drug, due to SOL’s precipitation stability and solubility enhancing properties. The presence of strong molecular interactions between the API and the selected matrix/carrier was suggested and verified both experimentally and theoretically, and was considered as a crucial mechanism for the physical stability and sustained supersaturation of the prepared ASDs.

## Figures and Tables

**Table 1:** Estimation of HSPs for DRN based on the Hoftyzer−Van Krevelen group contribution method.

Structural Group	N	N·F_di_ (J^1/2^ cm^3/2^ mol^−1/2^)	N·F_pi_^2^ (J cm^3^ mol^−2^)	N·E_hi_ (J mol^−1^)
−CH_3_	4	1680	0	0
−CH_2_−	12	3240	0	0
=C<	2	140	0	0
−CO−	1	290	770	2000
−O−	2	200	320,000	6000
−N<	1	20	800	5000
−NH−	1	160	210	3100
Benzyl ring	2	2860	24,200	0
−SO_2_	1	1129	1,844,164	11,670
	*δ_t_* (MPa^1/2^)	*δ_d_* (MPa^1/2^)	*δ_p_* (MPa^1/2^)	*δ_h_* (MPa^1/2^)
	18.5	16.6	3.6	7.3

## Data Availability

The data presented in this study are available upon request from the corresponding author.

## References

[1] Zareba K.M. (2006). Dronedarone: A new antiarrhythmic agent. Drugs Today.

[2] Jardan Y.A.B., Brocks D.R. (2016). The pharmacokinetics of dronedarone in normolipidemic and hyperlipidemic rats. Biopharm. Drug Dispos..

[3] Hohnloser S.H., Crijns H.J., van Eickels M., Gaudin C., Page R.L., Torp-Pedersen C., Connolly S.J. (2009). Effect of dronedarone on cardiovascular events in atrial fibrillation. N. Engl. J. Med..

[4] Touboul P., Brugada J., Capucci A., Crijns H.J., Edvardsson N., Hohnloser S.H. (2003). Dronedarone for prevention of atrial fibrillation: A dose-ranging study. Eur. Heart J..

[5] Han S.D., Jung S.W., Jang S.W., Jung H.J., Son M., Kim B.M., Kang M.J. (2015). Preparation of solid dispersion of dronedarone hydrochloride with Soluplus(^®^) by hot melt extrusion technique for enhanced drug release. Chem. Pharm. Bull..

[6] Marcolino A.I.P., Macedo L.B., Nogueira-Librelotto D.R., Fernandes J.R., Bender C.R., Wust K.M., Frizzo C.P., Mitjans M., Vinardell M.P., Rolim C.M.B. (2019). Preparation, characterization and in vitro cytotoxicity study of dronedarone hydrochloride inclusion complexes. Mater. Sci. Eng..

[7] Han S.D., Jung S.W., Jang S.W., Son M., Kim B.M., Kang M.J. (2015). Reduced Food-Effect on Intestinal Absorption of Dronedarone by Self-microemulsifying Drug Delivery System (SMEDDS). Biol. Pharm. Bull..

[8] Abramovici B., Gautier J.-C., Gromenil J.-C., Marrier J.-M. (2008). Solid Pharmaceutical Composition Containing Benzofuran Derivatives. U.S. Patent.

[9] Mahapatra A.K., Samoju S., Patra R.K., Pn M. (2014). Dissolution enhancement of dronedarone hydrochloride by complexation with β-CD and HP β-CD: Dissolution and physicochemical characterization. Thai J. Pharm. Sci..

[10] Kordić Š., Matijašić G., Gretić M. (2018). Prediction of particle size distribution of dronedarone hydrochloride in spiral jet mill using design of experiments. Chem. Eng. Commun..

[11] Kovvasu S.P., Kunamaneni P., Yeung S., Rueda J., Betageri G.V. (2019). Formulation of Dronedarone Hydrochloride-Loaded Proliposomes: In Vitro and In Vivo Evaluation Using Caco-2 and Rat Model. AAPS PharmSciTech.

[12] Jung H.J., Han S.D., Kang M.J. (2015). Enhanced Dissolution Rate of Dronedarone Hydrochloride via Preparation of Solid Dispersion using Vinylpyrrolidone-Vinyl Acetate Copolymer (Kollidone® VA 64). Bull. Korean Chem. Soc..

[13] Kapourani A., Palamidi A., Kontogiannopoulos K.N., Bikiaris N.D., Barmpalexis P. (2021). Drug Amorphous Solid Dispersions Based on Poly(vinyl Alcohol): Evaluating the Effect of Poly(propylene Succinate) as Plasticizer. Polymers.

[14] Chiou W.L., Riegelman S. (1971). Pharmaceutical Applications of Solid Dispersion Systems. J. Pharm. Sci..

[15] Pandi P., Bulusu R., Kommineni N., Khan W., Singh M. (2020). Amorphous solid dispersions: An update for preparation, characterization, mechanism on bioavailability, stability, regulatory considerations and marketed products. Int. J. Pharm..

[16] Tran T., Le L. Hybrid Backscatter and Underlay Transmissions in RF-Powered Cognitive Radio Networks. Proceedings of the 2019 26th International Conference on Telecommunications (ICT).

[17] Cai T., Zhu L., Yu L. (2011). Crystallization of Organic Glasses: Effects of Polymer Additives on Bulk and Surface Crystal Growth in Amorphous Nifedipine. Pharm. Res..

[18] DeBoyace K., Wildfong P.L.D. (2018). The Application of Modeling and Prediction to the Formation and Stability of Amorphous Solid Dispersions. J. Pharm. Sci..

[19] Diaz-Mora N., Zanotto E.D., Hergt R., Müller R. (2000). Surface crystallization and texture in cordierite glasses. J. Non Cryst. Solids.

[20] Hasebe M., Musumeci D., Powell C.T., Cai T., Gunn E., Zhu L., Yu L. (2014). Fast Surface Crystal Growth on Molecular Glasses and Its Termination by the Onset of Fluidity. J. Phys. Chem. B.

[21] Haser A., Zhang F. (2018). New Strategies for Improving the Development and Performance of Amorphous Solid Dispersions. AAPS PharmSciTech.

[22] Huang S., Williams R.O. (2018). Effects of the Preparation Process on the Properties of Amorphous Solid Dispersions. AAPS PharmSciTech.

[23] Kawakami K. (2017). Supersaturation and crystallization: Non-equilibrium dynamics of amorphous solid dispersions for oral drug delivery. Expert Opin. Drug Deliv..

[24] Wu T., Sun Y., Li N., de Villiers M.M., Yu L. (2007). Inhibiting Surface Crystallization of Amorphous Indomethacin by Nanocoating. Langmuir.

[25] Wu T., Yu L. (2006). Surface Crystallization of Indomethacin Below Tg. Pharm. Res..

[26] Baghel S., Cathcart H., O’Reilly N.J. (2016). Polymeric Amorphous Solid Dispersions: A Review of Amorphization, Crystallization, Stabilization, Solid-State Characterization, and Aqueous Solubilization of Biopharmaceutical Classification System Class II Drugs. J. Pharm. Sci..

[27] Li M., Meng F., Tsutsumi Y., Amoureux J.-P., Xu W., Lu X., Zhang F., Su Y. (2020). Understanding Molecular Interactions in Rafoxanide–Povidone Amorphous Solid Dispersions from Ultrafast Magic Angle Spinning NMR. Mol. Pharm..

[28] Tran T.T.D., Tran P.H.L. (2020). Molecular Interactions in Solid Dispersions of Poorly Water-Soluble Drugs. Pharmaceutics.

[29] Baird J.A., Van Eerdenbrugh B., Taylor L.S. (2010). A classification system to assess the crystallization tendency of organic molecules from undercooled melts. J. Pharm. Sci..

[30] Tung N.-T., Tran C.-S., Nguyen T.-L., Pham T.-M.-H., Chi S.-C., Nguyen H.-A., Bui Q.-D., Bui D.-N., Tran T.-Q. (2021). Effect of surfactant on the in vitro dissolution and the oral bioavailability of a weakly basic drug from an amorphous solid dispersion. Eur. J. Pharm. Sci..

[31] Koromili M., Kapourani A., Barmpalexis P. (2023). Preparation and Evaluation of Amorphous Solid Dispersions for Enhancing Luteolin&rsquo;s Solubility in Simulated Saliva. Polymers.

[32] Engers D., Teng J., Jimenez-Novoa J., Gent P., Hossack S., Campbell C., Thomson J., Ivanisevic I., Templeton A., Byrn S. (2010). A solid-state approach to enable early development compounds: Selection and animal bioavailability studies of an itraconazole amorphous solid dispersion. J. Pharm. Sci..

[33] Kapourani A., Vardaka E., Katopodis K., Kachrimanis K., Barmpalexis P. (2019). Rivaroxaban polymeric amorphous solid dispersions: Moisture-induced thermodynamic phase behavior and intermolecular interactions. Eur. J. Pharm. Biopharm..

[34] Li D., Panchal K., Mafi R., Xi L. (2018). An Atomistic Evaluation of the Compatibility and Plasticization Efficacy of Phthalates in Poly(vinyl chloride). Macromolecules.

[35] van Gunsteren W.F., Mark A.E. (1998). Validation of molecular dynamics simulation. J. Chem. Phys..

[36] Koromili M., Kapourani A., Koletti A., Papandreou G., Assimopoulou A.N., Lazari D., Barmpalexis P. (2022). Preparation and Evaluation of Siderol Amorphous Solid Dispersions: Selection of Suitable Matrix/Carrier. AAPS PharmSciTech.

[37] Verma S., Rudraraju V.S. (2014). A Systematic Approach to Design and Prepare Solid Dispersions of Poorly Water-Soluble Drug. AAPS PharmSciTech.

[38] Greenhalgh D.J., Williams A.C., Timmins P., York P. (1999). Solubility parameters as predictors of miscibility in solid dispersions. J. Pharm. Sci..

[39] Marom E., Rubnov S. (2012). Amorphous Form of. Dronedarone. Patent.

[40] Obata T., Suzuki Y., Ogawa N., Kurimoto I., Yamamoto H., Furuno T., Sasaki T., Tanaka M. (2014). Improvement of the antitumor activity of poorly soluble sapacitabine (CS-682) by using Soluplus^®^ as a surfactant. Biol. Pharm. Bull..

[41] Zi P., Zhang C., Ju C., Su Z., Bao Y., Gao J., Sun J., Lu J., Zhang C. (2019). Solubility and bioavailability enhancement study of lopinavir solid dispersion matrixed with a polymeric surfactant—Soluplus. Eur. J. Pharm. Sci..

[42] Ogawa N., Hiramatsu T., Suzuki R., Okamoto R., Shibagaki K., Fujita K., Takahashi C., Kawashima Y., Yamamoto H. (2018). Improvement in the water solubility of drugs with a solid dispersion system by spray drying and hot-melt extrusion with using the amphiphilic polyvinyl caprolactam-polyvinyl acetate-polyethylene glycol graft copolymer and d-mannitol. Eur. J. Pharm. Sci..

[43] Zhang Y., Liu Y., Luo Y., Yao Q., Zhong Y., Tian B., Tang X. (2016). Extruded Soluplus/SIM as an oral delivery system: Characterization, interactions, in vitro and in vivo evaluations. Drug Deliv..

[44] Zhu C., Gong S., Ding J., Yu M., Ahmad E., Feng Y., Gan Y. (2019). Supersaturated polymeric micelles for oral silybin delivery: The role of the Soluplus–PVPVA complex. Acta Pharm. Sin. B.

